# A novel approach to non-segmented flow analysis. Part 1: An experimental system

**DOI:** 10.1155/S146392468700035X

**Published:** 1987

**Authors:** D. J. Malcolme-Lawes, G. A. Milligan, R. Newton

**Affiliations:** 1 Centre for Research in Analytical Chemistry and Instrumentation King's College London Strand London WC2R 2LS UK; 2 Biotech Instruments Ltd Camford Way Luton UK


Journal of Automatic Chemistry/*Journal of Clinical Laboratory Automation*, Vol. 9, No. 4 (October–December 1987), pp. 179–183

### A novel approach to non-segmented flow analysis
### Part 1: An experimental system

**D. J. Malcolme-Lawes, G. A. Milligan**
*Centre for Research in Analytical Chemistry and Instrumentation, King's College London, Strand, London WC2R 2LS*

**and R. Newton**
*Biotech Instruments Ltd, Camford Way, Luton, UK*

*An experimental non-segmented continuous-flow analyser is described, based on gas-pressure driven carrier liquid and reagents controlled by a series of computer-switched solenoid valves. Examples of the typical results and reproducibility obtained using a standard analytical procedure for phosphate determination are discussed.*

### Introduction

One of the most popular approaches to continuous-flow analysis in recent years has been the technique of flow-injection analysis (FIA) [1–3]. In essence, FIA relies on the mixing of a sample with specific reagents in a continuously flowing stream of carrier liquid while travelling to a detector. The detector monitors some property of the liquid which undergoes a change according to the extent of the selective reaction, which has occurred between the injection of the sample and its arrival at the detector. The principal advantage of this technique over the older, air-segmented continuous-flow analyser approach [4] is that the incompressibility of the non-segmented flow at the pressures commonly used in FIA (typically a few p.s.i.) allows the time interval between injection and detection to be reproduced with high accuracy. This, in turn, allows reaction products to be monitored without waiting for a steady-state to be reached, so permitting a high rate of sampling to be achieved and allowing chemistries in which reactions do not go to completion to be employed.

A simple FIA manifold is illustrated in figure 1. The system may be realized by injection of a sample aliquot into carrier stream containing reagents (normal FIA), or by injection of reagents into a flow containing the sample (inverse FIA). Both approaches result in limited flexibility because systems tend to be restricted to single reagent mixtures and single analytes.

A novel instrument has been developed which performs non-segmented flow analysis. The design objective was to retain the advantages of FIA (highly reproducible timing, so that reactions need not go to completion before the detection process), while discarding those aspects of the technique which seem undesirable (peristaltic pump tubing and limited flexibility in handling multiple analytes and reagents). In this paper an experimental version of the instrument is described. Some aspects of its

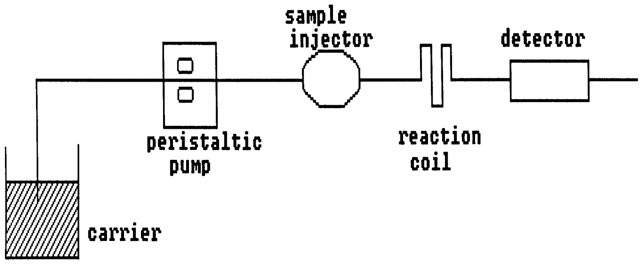

*Figure 1. Schematic representation of the conventional manifold used for FIA.*

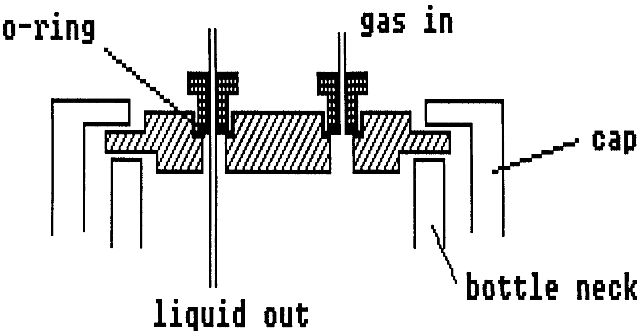

*Figure 2. Structure of pressurized reservoir caps. The connecting tubing may be 1/16th in PTFE.*

performance are discussed and some example results obtained using a standard analysis for phosphate are presented.

### The analyser

Essentially the instrument uses a carrier flow of a low cost, miscible but inert (in the sense that it does not contribute to the reaction) liquid, into which both sample and reagents are injected through computer-controlled valves. Early in the design of the analyser we decided against the use of peristaltic pumps in favour of gas pressure propulsion of the carrier flow. This has the advantages of producing a liquid flow which gives rise to less noise in the detector signal than can be obtained from peristaltic pumps, and allows PTFE tubing to be used throughout the system – which in turn allows a wide range of solvents and carriers to be employed.

The carrier and flushing liquids and the reagents were stored separately in Schott Duran bottles (generally 1000 and 500 ml capacities, respectively), fitted with closures fabricated from PTFE and held in place by the normal bottle cap through which a 30 mm hole had been bored. Two PTFE tubes were connected to each reservoir using Cheminert couplings, as illustrated in figure 2. One tube carried the pressurizing gas and terminated at a hole in the closure. The other tube carried the liquid from the reservoir and passed through the closure, the tube-closure seal being completed by a small silicone O-ring.

Carrier, flushing liquid, reagent and sample flows were controlled using solenoid valves connected together as a manifold using 1/16th in PTFE tubing. Solenoid valves

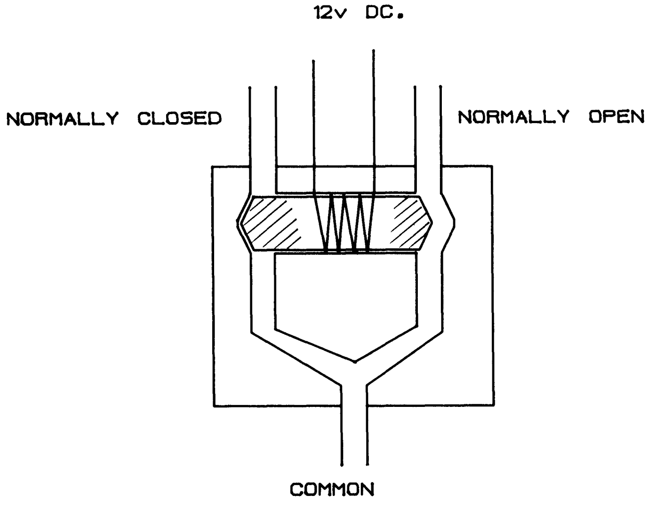

*Figure 3. Structure of the Lee solenoid valves used in the experimental instrument. The shaded plunger moves to the right when current passes through the coil.*

have previously been used for sample injection [5], although not, as far as we are aware, for reagent mixing and carrier control in the automated manner described here. For this experimental model of the analyser Lee valves (type LFYX0500200AB) by 12 V logic levels were used. The structure of this valve is shown schematically in figure 3 and operation consists essentially of closing one of the two pathways between the common connection (C) and the others, normally open (NO) and normally closed (NC). The normally open channel is open unless current passes through the solenoid, in which case the actuator moves to the right, closing the NO channel and leaving the NC channel open.

The valve connection manifold is shown in figure 4. For this experimental instrument a limit of four reagent reservoirs was set by the number of valves available, although there is no reason why a much larger number could not be employed. Valves 1–4 allow selected reagent to enter and fill the section between valves 5 and 6, and the four valves were equidistant from the mixing point (valve 5) in order to simplify injection timing. The sample loop may be filled by needle injection, suction or any other automated technique. Once the reagent or reagents have filled the section between valve 5 and 6, then valve 5 is closed (along with any reagent valves previously opened) and the sample loop is switched in. At this point the pathway between valve 5 and 6 contains a reagent–sample–reagent sandwich of precisely reproducible volumes, and closure of valves 0, 6, 5 and 7 causes this sandwich to be propelled by carrier through valve 7 to the detector. During this passage, sample/reagent mixing occurs and reaction product may be formed. Once the absorbance of the reaction mixture has been measured valves 5 and 6 may be opened and the pathway between flushed by wash liquid (which may be of the same composition as the carrier), which also flushes the reagent valves and interconnecting tubes.

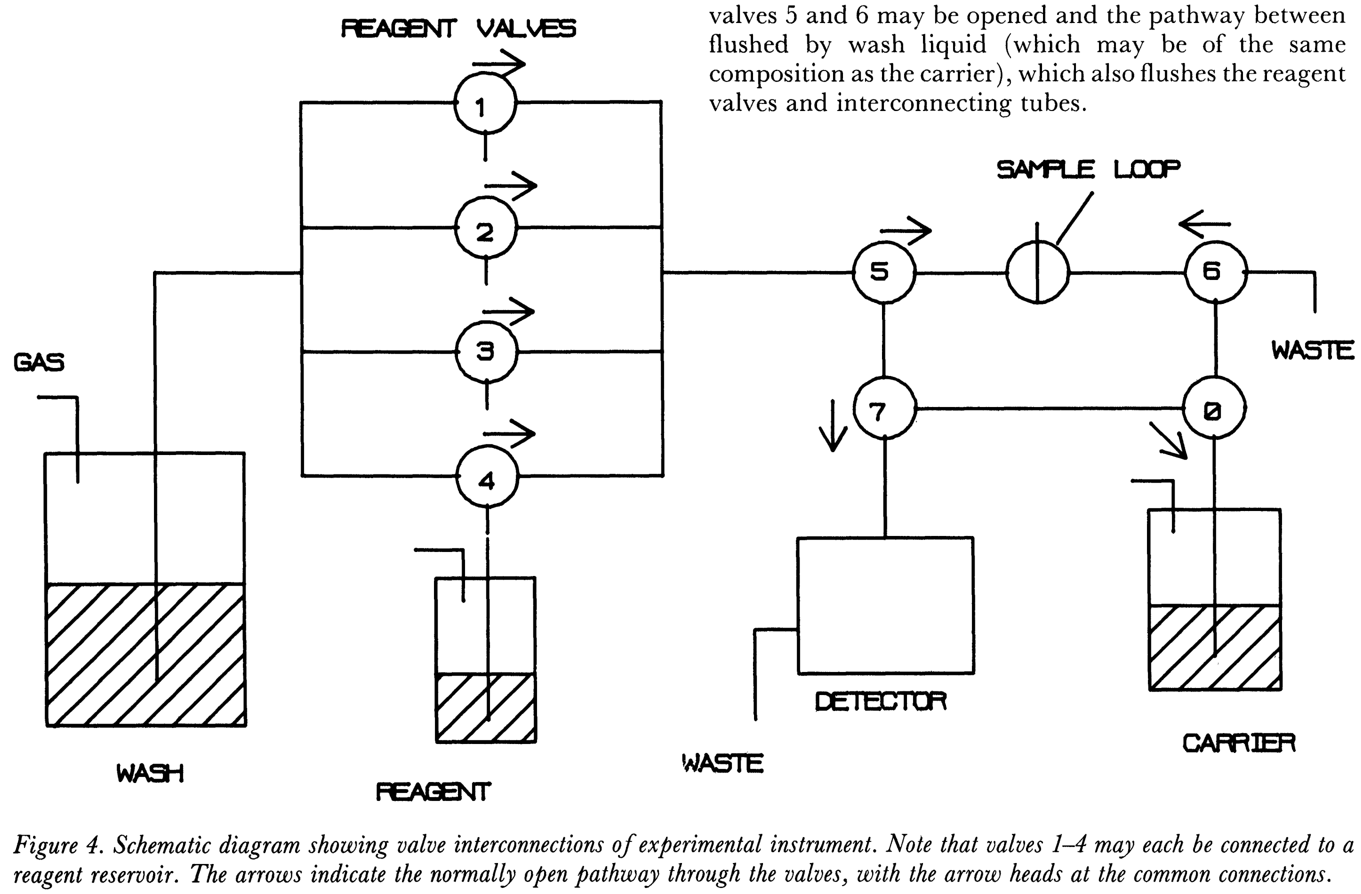

*Figure 4. Schematic diagram showing valve interconnections of experimental instrument. Note that valves 1–4 may each be connected to a reagent reservoir. The arrows indicate the normally open pathway through the valves, with the arrow heads at the common connections.*

*Table 1. Valve configurations during analysis.*

| Operation | Valve pattern 0 | 1 | 2 | 3 | 4 | 5 | 6 | 7 |
|---|---|---|---|---|---|---|---|---|
| Default/flush | NO | NO | NO | NO | NO | NO | NO | NO |
| Reagent load | NO | (User selected) | | | | NO | NO | NO |
| Awaiting sample | NC | NO | NO | NO | NO | NC | NO | NO |
| Analyse | NC | NO | NO | NO | NO | NC | NC | NC |
| Stop flow | NC | NO | NO | NO | NO | NO | NO | NO |

The sequence of valve operations is summarized in table 1, in which NO refers to the normally open state of a valve and is the condition when it is logically off. The 'awaiting sample' condition (which halts flow through valve 5 and the detector) is required only for manual sample loading, where there may be a significant delay between reagent assembly and the operator actually loading the sample. For manual sample injection, the operation of introducing the sample into the reagent sandwich is sensed by an optical sensor attached to the injection valve. This allows the computer to start the timing sequence for the remainder of the analysis – which may require immediate switching to the 'analysis' state.

The only important volume, and hence length of interconnecting tubing, is for the segment between valves 5 and 6 – and, of course, the sample loop volume. There are two parts to this segment, one either side of the sample loop, and for maximum sensitivity these must be large enough to ensure that there is always an excess of reagent over sample as the two mix together and travel to the detector. On the other hand, making these volumes small allows for considerable economy in the use of reagents. For the purposes of this instrument it was assumed that the user would want a compromise between the sensitivity (related to peak height) and sample throughput rate (inversely proportional to peak width), and the height-to-width ratio of detected peaks was taken as suitable criterion for parameter optimization. It was found that, using a flow rate of 1·5 ml/min, a maximum value of the height-to-width ratio was obtained for a sample loop volume of 175 μl, with volumes of 40 and 140 μl for the volumes of tubing on the valve 5 and valve 6 side of the loop respectively. Of course, the instrument could be optimized on other criteria to achieve higher sensitivity or higher sample throughput by using different volumes.

For the prototype analyser a spectrophotometric detector was chosen, as this allowed many classical reactions to be used relatively easily – much as in conventional flow injection analysis [6]. Two detectors have been used to date, one a filter colorimetric detector (Biotech Instruments Ltd, Luton, UK), and the other a Cecil UV-Vis spectrometer (Model CE 5095, Cecil Instruments Ltd, Cambridge, UK) fitted with a 100 μl flow cell.

The instrument was connected to a Commodore SX64 microcomputer (Commodore Business Machines Ltd, Slough, UK) using a multifunction interface unit shown in outline in figure 5. The output from the detector was monitored using a dynamic analogue interface [7], which

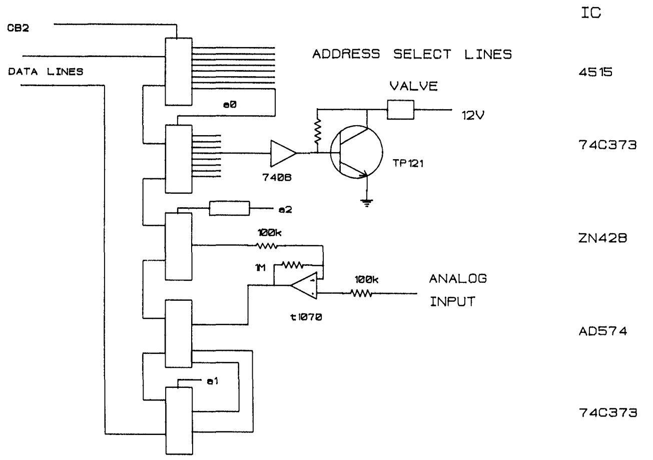

*Figure 5. Principal elements of the computer interface. The address decoder, valve switching logic buffers (followed by Darlington boosters), and the analogue input circuits may all be connected to a microcomputer parallel port.*

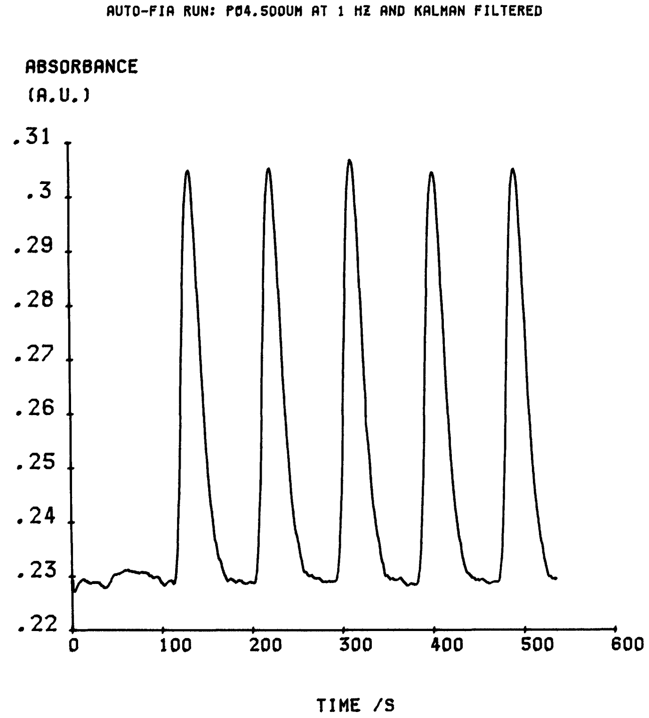

*Figure 6. Peaks recorded following one injection of water (blank) and five injections of sample containing phosphate (approximately 500 μM). See text for details.*

allowed a reading over the range 0–1 V with a precision of approximately 10 μV. The valve operation was controlled by a logic output interface which operated TP121 transistors in series with the valve solenoids. The sample-loaded sensor was connected to the computer's paddle port, where the change in resistance of a photodiode forming part of standard slotted opto-switch (RS Components) as the loop valve turned was monitored.

*Instrument performance*

The reproducibility of absorbance peaks recorded with this instrument is largely governed by the precision of the timing of the valve operations. Figure 6 shows the detector output recorded from five successive samples of phosphate (approximately 500 μM) monitored using the colour developed by the reaction with a mixture of sodium molybdate ($3{\cdot}2 \times 10^{-2}$ M) in 0·4 M nitric acid, and ascorbic acid ($2{\cdot}7 \times 10^{-3}$ M). The absorbance was monitored at 500 nm and carrier and wash liquids were both distilled water. It should be noted that the absorbance axis in figure 6 has not been corrected for the arbitrary (manual) setting of the base-line position.

Clearly the instrument gives reproducible results and the sensitivity is of the same order as one would expect from conventional flow-injection analysis. However, the technique used does give rise to two features which become superimposed on the detector base-line. The first arises from the refractive index change brought about by the introduction of reagent into the carrier flow. This results in a lens-like liquid–liquid interface which focuses the light passing through the flow cell more effectively than a homogeneous liquid [8]. The net result is an increase in transmission and a negative-going peak (or trough) on the absorbance record as the reagent–sample–reagent sandwich enters the flow cell. A smaller positive-going

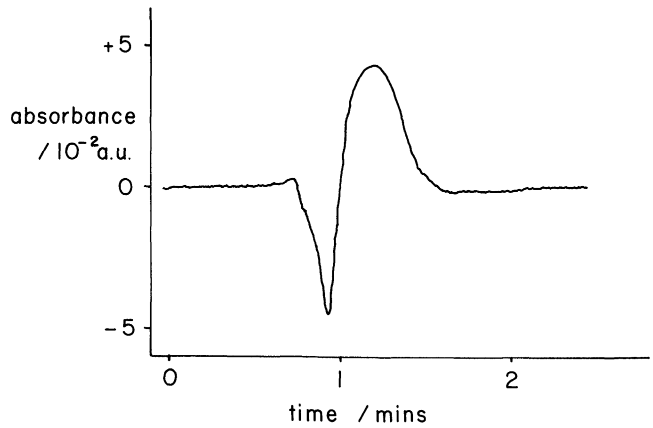

*Figure 7. A deliberately extreme example of the base-line fluctuation caused by the introduction of a strong reagent (5 M NaOH) into a distilled water carrier.*

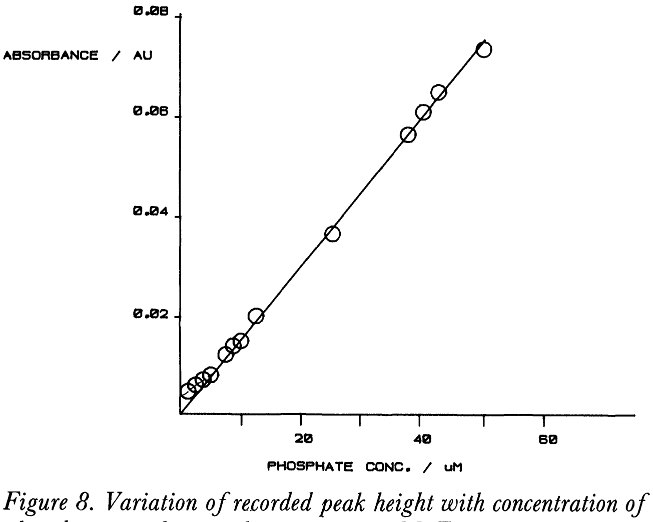

*Figure 8. Variation of recorded peak height with concentration of phosphate samples over the range 1–50 μM. For reagent details see description of figure 6.*

peak may be produced by a lens at the tail end of the sandwich, although as this has travelled a greater distance and passed through the sample valve this lens is more diffuse and the effect smaller. Because many reagents will have a different absorbance from the carrier liquid at the monitoring wavelength, the passage of reagent through the cell may also contribute to a positive going absorbance signal immediately following the trough.

These effects can be clearly seen in figure 7, the absorbance monitored at 500 nm from the passage of an aliquot of 5 M NaOH through the flow cell – an extreme example. In this case both the trough and the peak give rise to transmission changes of the order of 0·04 AU. In many cases, particularly where reagents are not so concentrated, reaction product absorbances may be much larger than the disturbances which arise from these sources. In such cases the monitored peak height is found to vary linearly with sample concentration over a substantial range of sample sizes. However, at low sample concentration these effects do give rise to a deviation from linearity. Figure 8 shows the variation of peak height (in AU) with sample concentration for a series of analyses performed on samples of phosphate, using the reagents and monitoring wavelength described above. Significant deviations from linearity are apparent below $7 * 10^{-6}$ M [$PO_4^{3-}$]. Fortunately such deviations can be removed by subtracting the signal recorded from a blank, which is relatively simple to arrange using the computer's software.

Even without such corrections, the sensitivity of the experimental instrument is high, approaching or exceeding the sensitivities of conventional flow analysis instruments [9], so it is interesting to consider the potential benefits of this approach compared with more conventional systems. At first sight the instrument may appear considerably more complex than an FIA system of the kind shown in figure 1. However, while basic instruments such as the FIAstar (Tecator AB, Hoganas, Sweden) are available, most commercial instruments, such as the Tecator FIA 5020 (Tecator AB), have themselves become considerably more complex than a diagram of the liquid flow path suggests, incorporating items such as microprocessor controlled four-way valves and multiple eight-roller peristaltic pumps. As a result, the cost of components for a commercial FIA system can easily exceed that for our multi-valve design. Furthermore, the system described here is amenable to relatively inexpensive expansion to allow a larger number of reagents to be handled, so that sequential analysis for several different analytes is easily accomplished. (An expanded version of the instrument incorporating eight reagent bottles is under construction.)

The use of an easily programmable microcomputer for total control of the system has particular attractions in that, when coupled with an autosampler, the system may be programmed to calibrate itself from a set of standards, and to analyse multiple analytes sequentially from many samples whilst running unattended. Probably the three main attractions of the new approach are the low consumption of reagents (typically less than 400 μl per analysis), the ability to prepare reagents *in situ* from separate components (allowing reagents of limited stability to be employed), and the ability to handle a wide range of organic solvents. These features coupled with the high reliability and reproducibility of the experimental system, suggest that this new approach may offer significant benefits for routine analysis.

### Acknowledgements

This work was performed with the support of the Science and Engineering Research Council. Patent protection has been applied for to cover some aspects of the instrument described in this paper. D.J.M-L. is a Royal Society Research Fellow in the Physical Sciences.

### References

1. Stewart, K. K., *Talanta,* **28** (1981), 789.
2. Ruzicka, J. and Hansen, E. H., Flow Injection Analysis, in *Chemical Analysis,* Vol. 62. (J. Wiley, New York, 1981).
3. Valcarcel, M. and Luque de Castro, M. D., *Flow Injection Analysis Principles and Applications* (Ellis Horwood Ltd, Chichester, 1987).
4. Skeggs, T., *American Journal of Clinical Pathology,* **28** (1957), 311.
5. Harrow, J. J. and Janata, J., *Analytical Chemistry,* **55** (1983), 2461.
6. *Flow Injection Analysis Bibliography* (Tecator Ltd, 1985).
7. Malcolme-Lawes, D. J., *Laboratory Microcomputer* (in press).
8. Vickrey, T. M., *Liquid Chromatography Detectors* (Marcel Dekker Inc., New York, 1983).
9. Application subnote 62-01/83, Tecator AB, Hoganas, Sweden (1983).

### 36TH PITTSBURGH CONFERENCE AND EXPOSITION (22–26 FEBRUARY 1988) IN NEW ORLEANS: AWARDS SCHEDULED

Professor Henry Freiser has been named by the Society for Analytical Chemists of Pittsburgh to receive the **1988 Pittsburgh Analytical Chemistry Award**. Professor Freiser was one of the early founders of the Pittsburgh Conference. He has been a leading and influential educator in analytical chemistry and has also contributed significantly to the science and profession of analytical chemistry. His research in metal chelate chemistry, solvent extraction, processes, and ion selective electrodes is widely known and respected. Professor Freiser will receive his award at the award symposium on Tuesday, February 23.

Professor K. Narahari Rao will receive the **1988 Pittsburgh Spectroscopy Award**, sponsored by the Spectroscopy Society of Pittsburgh. Professor Rao is being honored for his numerous and wide-ranging contributions to the field of high resolution infrared spectroscopy. The award will be presented to Dr Rao, who is currently Professor of Physics at Ohio State University, by Ernest F. Tretow, SSP Chairman, at a special symposium on Wednesday, February 24.

The **Dal Nogare Award** will be presented by The Chromatography Forum of the Delaware Valley to Dr Harold Walton, professor emeritus and senior research associate of the Cooperative Institute for Research in Environmental Sciences in Boulder, Colorado. Dr Walton has received the award for his achievements in the field of chromatography, particularly for his contributions in the study of ligand-exchange and ion exchange separations, both theoretical precepts and practical aspects. The Dal Nogare Award has been given annually since 1972 in recognition of scientists who have contributed significantly to the understanding and practice of chromatography. The award will be presented at an award symposium on Tuesday, February 23.

Professor Royce Murray of the University of North Carolina is the fifth recipient of the *C. N. Reilley Award* in Electroanalytical Chemistry. Professor Murray is being recognized for his extensive achievements in the area of modified electrodes, particularly as amperometric chemical sensors. The award is administered by the Society for Electroanalytical Chemistry and is sponsored by Bioanalytical Systems, Inc., and the Reilley Endowment Fund of SEAC. The award symposium will be held on Wednesday, February 24.

The **1988 Williams-Wright Award** will be presented to Dr Darwin L. Wood of AT & T Bell Laboratories. The award is sponsored by the Coblentz Society and is presented annually to a person who has made significant contributions to vibrational spectroscopy while working in industry. One of the specialties of this year's awardee is the chemistry of processes for making fibre optical materials. The award will be presented at a symposium on Tuesday, February 23.

The **Keene P. Dimick Award** for Analytical Chemistry by Gas Chromatography will be presented for the first time at the 1988 Pittsburgh Conference. This award, sponsored by Dr Keene P. Dimick, will be presented at a symposium on the Thirty-year Anniversary of Capillary (Gas) Chromatography on Monday, February 22. The winner will be announced in the Preliminary Program.

The **Tomas Hirschfeld Award** in Near-Infrared Analysis, an award sponsored by Bran+Luebbe/Technicon Industrial Systems, will be presented for the second time at the 1988 Pittsburgh Conference to a graduate student working in the field of near-infrared analysis. The award will be presented in a session of contributed papers on near-infrared analysis on Monday, February 22. The winner will be announced in the Preliminary Program.

